# Modified RNA triplexes: Thermodynamics, structure and biological potential

**DOI:** 10.1038/s41598-018-31387-5

**Published:** 2018-08-29

**Authors:** Marta Szabat, Elzbieta Kierzek, Ryszard Kierzek

**Affiliations:** Institute of Bioorganic Chemistry, Polish Academy of Sciences, Noskowskiego 12/14, 61-704 Poznan, Poland

## Abstract

The occurrence of triplexes *in vivo* has been well documented and is determined by the presence of long homopurine-homopyrimidine tracts. The formation of these structures is the result of conformational changes that occur in the duplex, which allow the binding of a third strand within the major groove of the helix. Formation of these noncanonical forms by introducing synthetic triplex-forming oligonucleotides (TFOs) into the cell may have applications in molecular biology, diagnostics and therapy. This study focused on the formation of RNA triplexes as well as their thermal stability and biological potential in the *HeLa* cell line. Thermodynamics studies revealed that the incorporation of multiple locked nucleic acid (LNA) and 2-thiouridine (2-thioU) residues increased the stability of RNA triplexes. These data suggest that the number and position of the modified nucleotides within TFOs significantly stabilize the formed structures. Moreover, specificity of the interactions between the modified TFOs and the RNA hairpin was characterized using electrophoretic mobility-shift assay (EMSA), and triplex dissociation constants have been also determined. Finally, through quantitative analysis of GFP expression, the triplex structures were shown to regulate GFP gene silencing. Together, our data provide a first glimpse into the thermodynamic, structural and biological properties of LNA- and 2-thioU modified RNA triplexes.

## Introduction

The biological functions of nucleic acids are determined by the conformational alterations from one structure to another. The polymorphic nature of DNA and RNA allows them to adopt a variety of structures depending on the sequences, the presence of ions and other biomolecules and the local environment. The formation of noncanonical DNA and RNA forms has been well documented and three-stranded nucleic acids are examples of these unusual structures.

Triplexes were first observed by Felsenfeld *et al*. and occur when a triplex-forming oligonucleotide (TFO) binds specifically in the major groove of DNA or RNA duplexes^1^. These structures are classified into two main categories depending on the TFO base composition and binding orientation relative to its target site in the helix. The purine-rich strand of the duplex is recognized by a purine TFO through reverse Hoogsteen hydrogen bonds to form an antiparallel triplex (also called a purine motif). In the pyrimidine motif, a TFO consisting of cytosine and thymine residues binds parallel to a purine-rich strand of helical DNA or RNA via Hoogsteen hydrogen bonds. Pyrimidine oligonucleotides require base protonation and form triplexes largely in a pH-dependent manner. Because the recognition of DNA or RNA duplexes through an unmodified third strand is typically limited to the presence and length of homopurine-homopyrimidine tracts and low pH conditions, it is important to enhance the biological stability of the triplexes and increase TFO binding affinity toward the complementary strand of the duplex. Therefore, various chemically modified oligonucleotides have been developed to improve the properties of a triple-helical structure and its potential as a molecular tool for applications under physiological conditions. The examples of modifications that stabilize triplexes are LNAs^2^, 2′-O-methyl-RNAs^3^, peptide nucleic acids (PNAs)^4^, 2-thiouridine^3^ and 5-methylcytidine^5^.

LNA is a conformationally constrained nucleotide analogue, allowing to increase the binding affinity, in comparison with DNA and RNA^6–8^. Thermal stability studies have shown that LNA residues enhance the thermodynamic stability and kinetic properties when introduced into duplexes^9–12^ and highly ordered structures (i.e. i-motif^13^, quadruplexes^14^, triplexes^3,15,16^). Recently, Zain’s group has confirmed that the number and position of LNA substitutions in TFOs influence the DNA triplex formation, by TFOs conformational pre-organization for major groove binding^16^. Furthermore, it has been reported that incorporation of LNA can shift the pK value of cytidine in TFO^17^. The beneficial influence on the stability, kinetics, resistance to nucleases and cellular uptake make LNAs interesting tools in triplex-based approaches^16–18^. Moreover, Chen and co-workers have reported that also 2-thioU in TFO is effective in terms of stabilization of RNA triplexes^3^.

A number of reports concern DNA or mixed RNA-DNA triplex structures formed under various conditions *in vitro* and *in vivo*^3,19,20^, whereas few studies have shown triplex formation by targeting RNA duplex regions. However, it has been found that RNA triplexes are formed mainly in functionally important RNAs, including telomerase RNA^21^, group I and II introns^22,23^, long noncoding RNAs^24^ and ribosomal RNAs^25^. Furthermore, it is known that endogenous mirror repeat DNA sequences form intramolecular triplexes, called the H-DNA structure, and play an important role *in vivo*^19^. Thus, triplexes of DNA and RNA have a great potential for applications in molecular biology, diagnostics and therapy^26,27^. Interaction between the TFO and DNA duplex could be a basis for antigene therapy, where a single oligonucleotide downregulates the target gene expression^28^. Moreover, the DNA triplex has been used in replication pausing, gene-targeted mutagenesis or genomic DNA mapping^29^. Interactions between noncoding RNA and genomic DNA are important in the biologically active RNA-dsDNA triplex^20^. However, some limitations of triplex approaches include the TFO base composition, specificity of TFO-duplex interactions and its pH-dependence.

In this paper, we present the triplex-forming thermodynamics and structural properties, binding affinity and cellular activity of RNA TFOs containing simultaneously LNA and 2-thioU modifications, for the first time. We have performed the thermodynamic analysis of the influence of multiple substitutions of LNA and 2-thioU derivatives on triplex thermal stability. The model RNA triplexes were characterized by a UV/VIS melting method and circular dichroism (CD) spectroscopy. In addition, the influence of modified nucleotides on the specificity of TFO-RNA hairpin interactions was determined. Lastly, the biological effect of RNA triplexes in a *HeLa* cell line was studied. LNA and 2-thioU residues cause an essential increase in triplex thermodynamic stability, however, the CD spectra show that these modifications do not change the overall structure of RNA triplexes. In addition, the TFO specificity and hairpin interaction studies indicated that LNA and 2-thioU residues are beneficial for triplex formation under the conditions similar to physiological ones. Notably, data from *HeLa* cells experiments revealed that TFOs containing LNAs and 2-thioUs can be alternative chemical tools for the regulation of gene expression in various triplex-based strategies.

## Results and Discussion

Some papers concerning triplex-based strategies revealed interesting and exceptional properties of these structures in therapeutic contexts^30–32^. TFOs constitute potential tools for downregulating gene expression, mediating gene-targeted mutagenesis and mapping genomic DNA^29^. Therefore, it is crucial to increase the thermodynamic stability of the triplexes under physiological conditions. LNAs appeared to be useful chemical tools to modulate the thermal stability of triple-helical structures^16,33^. Moreover, the incorporation of 2-thiouridine into the TFO strand has been shown to stabilize triplex formation with RNA and DNA duplex regions^3^. Although many studies have reported the functional properties of LNA and 2-thioU modified oligonucleotides in duplex and triplex^3,10,16,17^, the contributions of both modifications to triplex formation remains unknown. We expected that the multiple and simultaneous substitutions of LNA and 2-thioU within the TFO will induce its conformational changes, improve nuclease resistance and form more favorable binding of TFO to the RNA hairpin under neutral conditions^3,16^. Therefore, the studies of the effect of multiple LNA and 2-thioU substitutions on RNA triplex formation, followed by examination in GFP gene expression system in *HeLa* cells, were performed.

### The influence of simultaneous LNA and 2-thioU substitutions within the TFO strand on triplex thermal stability

**Figure 1:**
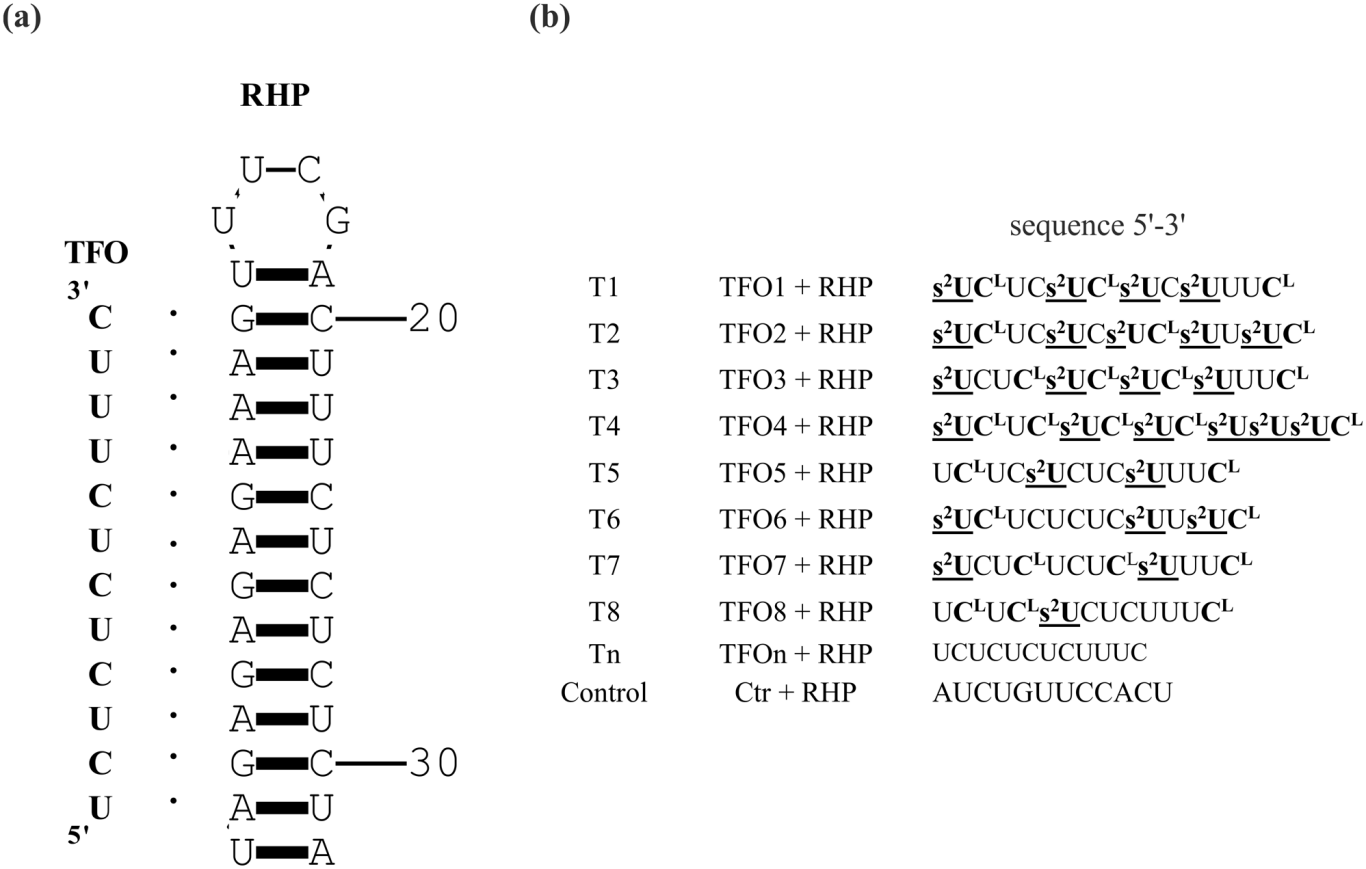
Schematic representation depicting the model RNA triplexes. (**a**) RNA triplex structure consists of a 12-mer TFO and 32-mer RNA hairpin (RHP). (**b**) Variants of RNA triplexes formed between unmodified TFO, called triplex Tn or modified TFOs containing LNA and 2-thioU moieties, triplexes T1–T8. Triplex Control was a referential probe in the *HeLa* cell line experiments.

First, we designed and synthesized unmodified and modified oligoribonucleotides to form nine variants of RNA triplexes (Fig. 1)^11^. Next, thermodynamic studies of the model RNA triplexes were conducted using the UV/VIS melting method. The thermodynamic data demonstrate that the simultaneous presence of LNA and 2-thioU modifications within the TFO strand has a significant influence on the thermal stabilities of T1–T8 model triplexes. The presence of LNA-cytidine and 2-thioU residues increased the melting temperatures of the RNA triplexes by 15.1 °C on average, in comparison to an unmodified counterpart (Table 1).

**Table 1:** Melting temperatures of model RNA triplexes (T_m1_) and RNA hairpins (T_m2_).

Name of RNA triplex	Melting temperatures		
	T_m1_ (°C)	T_m2_ (°C)	ΔT_m_ (°C)
Tn	46.4	79.3	—
T1	68.0	82.2	21.6
T2	67.1	82.5	20.7
T3	60.0	82.0	13.6
T4	64.1	81.2	17.7
T5	62.1	79.3	15.7
T6	53.2	80.9	6.8
T7	n.d.	(75)	n.d.
T8	56.0	81.4	9.6

Solution: 100 mM NaCl, 20 mM MES and 0.5 mM Na_2_EDTA, pH 7.0.

ΔT_m_ values were calculated on the basis of the difference between T_m1_ of T1–T8 triplexes and T_m1_ of Tn triplex.

As a result, we were unable to obtain reliable thermodynamic data for the T7 triplex due to the lack of two melting transitions (Supplementary Fig. S1). Moreover, as expected, the increases in melting temperature (ΔT_m_ parameter) were higher for T1, T2 and T4 triplexes (ΔT_m_ = 21.6, 20.7 and 17.7 °C, respectively), in comparison to T5, T6 and T8 triplexes (ΔT_m_ = 15.7, 6.8 and 9.6 °C, respectively). The changes in melting temperatures correspond to the numbers of LNA and 2-thioU residues within the TFO strand. This observation agrees with previous findings that showed the stabilization effect of LNA on triplex formation^16,34^. In general, the incorporation of LNA nucleotides pre-organizes the structure of TFO oligonucleotides and induces a *C3*′*-endo* conformation^10,12^. As a consequence, the interactions of the third strand containing LNA residues cause favorable energetic changes during the formation of triplexes. Recently it has been shown that the introduction of 2-thioU within the TFO increased the thermodynamic stability of the triplex^3^. The presence of a sulfur atom may improve the specificity of TFO interactions with the RNA hairpin by increasing the base stacking and van der Waals contacts^3^. In addition, most likely 2-thioU modification enhances TFO binding by reducing the thermodynamic cost of dehydration. Simultaneous incorporation of LNA and 2-thioU into a TFO strand leads to significant stabilization of the triplexes.

### Spectroscopic features of model triplexes

Structural conformation of model RNA triplexes was analyzed using CD spectroscopy. CD spectra provide information about the geometry of DNA and RNA structures and various types of tertiary interactions^35^. Previous papers revealed that the CD spectra of DNA triple-helical structures are characterized by a strong negative band at 212 nm^36,37^. To provide structural information on the model triple-helix, we have performed CD studies of nine isosequentional RNA triplexes, including unmodified Tn and modified T1–T8, containing multiple LNA and 2-thioU, under neutral conditions.

**Figure 2:**
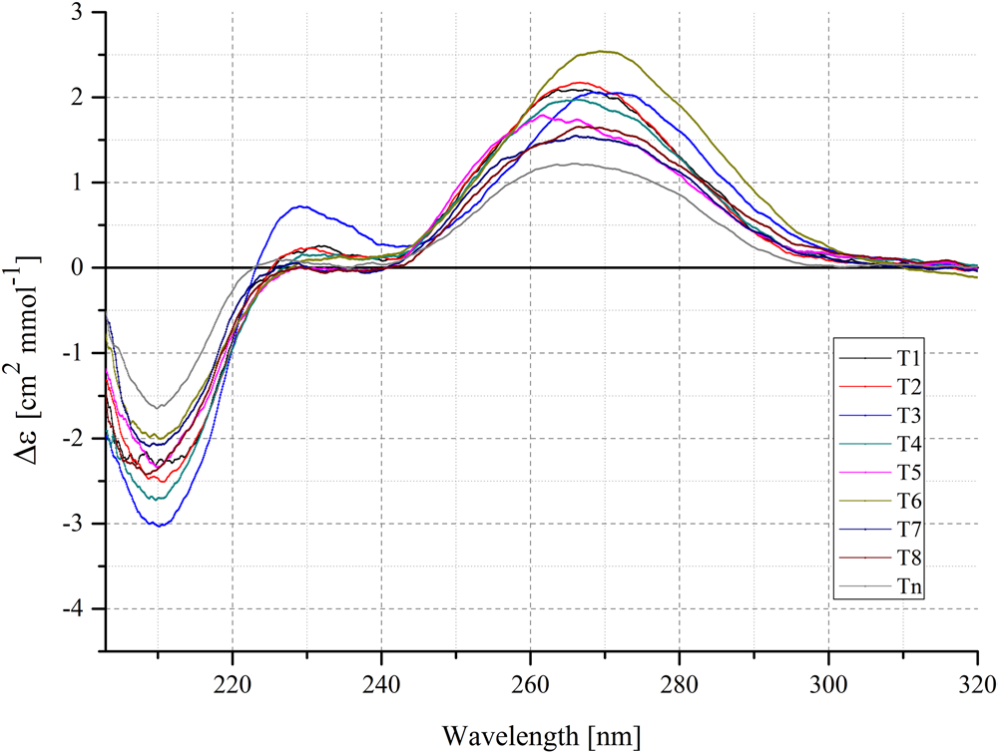
Circular dichroism spectra of modified T1–T8 triplexes in comparison to the unmodified Tn triplex at pH 7.0.

In this study, the CD spectra of the T1–T8 variants showed a CD profile indicating triplex formation with a high-amplitude negative band at approximately 212 nm and a strong positive band near 275 nm (Fig. 2). The CD measurements also revealed that the modified RNA triplexes show increased band intensity at 212 nm and 275 nm in comparison to the unmodified counterpart (Tn). This tendency is probably a result of conformational rearrangement, induced by the LNA and 2-thioU modified TFO, resulting in the formation of a stable triplex structure. This is in line with the previous reports regarding the conformational influences of LNA on TFO strand, and the enhanced binding of LNA-modified TFO to double-stranded target^16^.

### The binding affinity between the modified TFO and RNA hairpin

To gain insight into the specificity of TFO interactions with a RNA hairpin, we conducted an electrophoretic mobility shift assay (EMSA) and determined triplex dissociation constants under the conditions comparable to physiological ones. The gel mobility shift assay is a useful technique to quantitatively analyze triplex formation. The difference in charge density between double- and triple-helical structures leads to the retardation of duplex migration due to triplex formation^38^. The affinity of TFO variants to bind the model hairpin was investigated by electrophoresis experiments at pH 7.0. The value of the triplex dissociation constant (K_d_) was determined from the concentration of TFO that caused half of the hairpin to form a triplex^39^. In general, the binding affinity of TFO is strongly dependent on the pH, which is related to cytidine protonation. Based on the previous study, it is known that LNA substitution in the TFO shifts the pK of cytidine toward high values, promoting the triplex formation through Hoogsteen base pairing, at neutral pH^17^. In the present paper, the capacity of modified TFO to bind model RNA hairpin was investigated using the EMSA method. We have examined eight variants of the same TFO sequence, containing different number of LNA and 2-thioU substitutions. All of the studied TFOs formed triplex structures (T1–T8) at pH 7.0.

**Figure 3:**
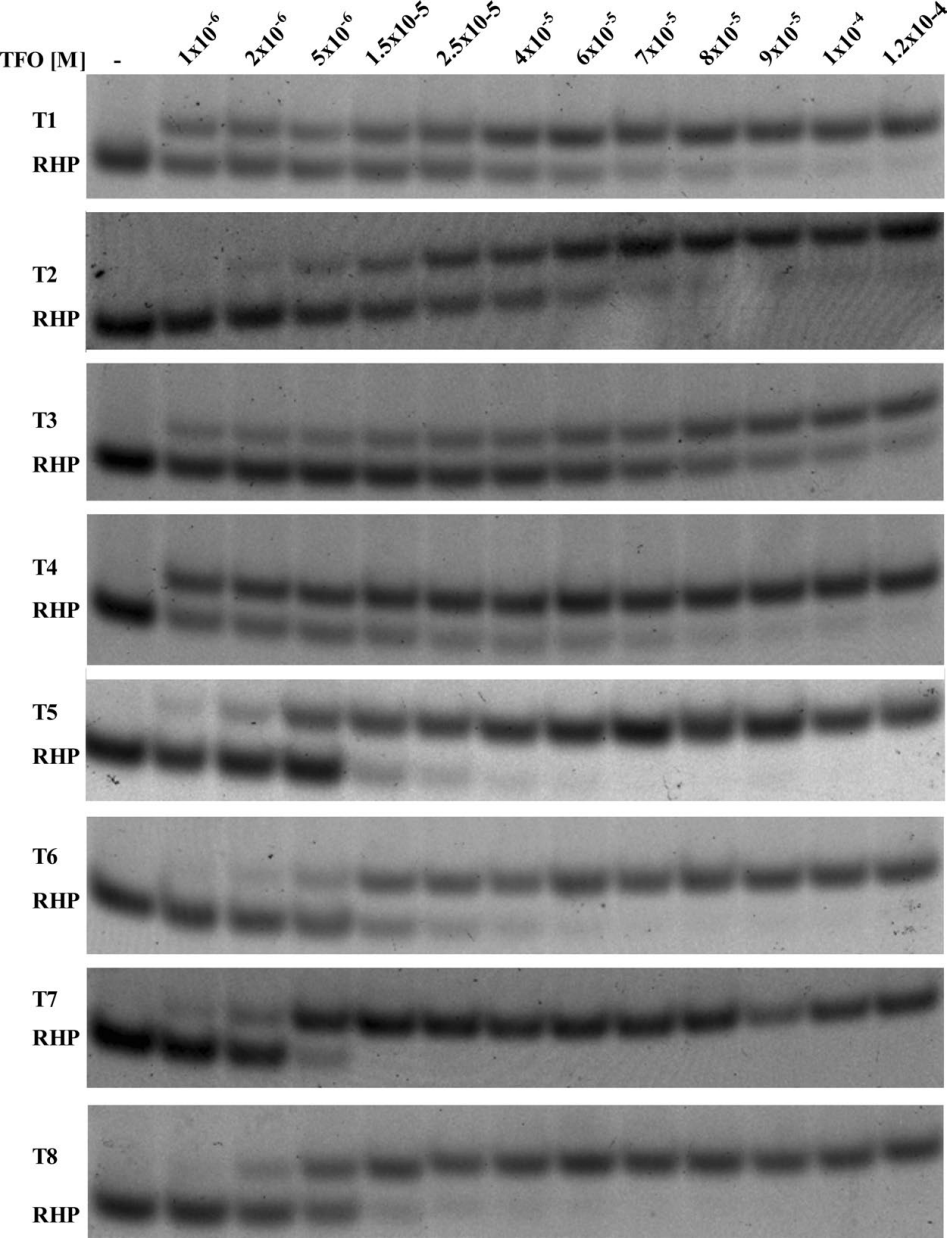
Electrophoretic mobility shift assays of model triplexes T1–T8 at pH 7.0.

Most reports in literature focus on the binding of TFO to DNA duplex^39–42^. Our results revealed that the binding affinities between the modified TFO and RNA hairpin are different among the model triplexes (Fig. 3). In our studies, the presence and number of LNA and 2-thioU nucleotides within the TFO strand have a significant influence on the value of the triplex dissociation constant (K_d_, Table 2). This is consistent with previous studies, which demonstrated that the number of LNA residues in TFO has a direct impact on the binding kinetics and triplex stability^8,34^. In addition, it has been shown that both target and TFO sequences directly affect the triplex formation and stability^2^.

**Table 2:** The value of dissociation constant (K_d_) of T1–T8 triplexes.

Triplex name	TFO sequences (5ʹ-3ʹ)												K_d_ ± ΔK_d_^*^ [µM]
Tn	U	C	U	C	U	C	U	C	U	U	U	C	no binding
T1	**s** ^**2**^ **U**	C^L^	U	C	**s** ^**2**^ **U**	C^L^	**s** ^**2**^ **U**	C	**s** ^**2**^ **U**	U	U	C^L^	3.01 ± 0.24
T2	**s** ^**2**^ **U**	C^L^	U	C	**s** ^**2**^ **U**	C	**s** ^**2**^ **U**	C^L^	**s** ^**2**^ **U**	U	**s** ^**2**^ **U**	C^L^	27.30 ± 7.10
T3	**s** ^**2**^ **U**	C	U	C^L^	**s** ^**2**^ **U**	C^L^	**s** ^**2**^ **U**	C^L^	**s** ^**2**^ **U**	U	U	C^L^	45.20 ± 9.04
T4	**s** ^**2**^ **U**	C^L^	U	C^L^	**s** ^**2**^ **U**	C^L^	**s** ^**2**^ **U**	C^L^	**s** ^**2**^ **U**	**s** ^**2**^ **U**	**s** ^**2**^ **U**	C^L^	2.47 ± 0.51
T5	U	C^L^	U	C	**s** ^**2**^ **U**	C	U	C	**s** ^**2**^ **U**	U	U	C^L^	7.60 ± 1.21
T6	**s** ^**2**^ **U**	C^L^	U	C	U	C	U	C	**s** ^**2**^ **U**	U	**s** ^**2**^ **U**	C^L^	9.54 ± 1.70
T7	**s** ^**2**^ **U**	C	U	C^L^	U	C	U	C^L^	**s** ^**2**^ **U**	U	U	C^L^	3.94 ± 0.47
T8	U	C^L^	U	C^L^	**s** ^**2**^ **U**	C	U	C	U	U	U	C^L^	5.20 ± 0.59

Solution: 100 mM NaCl, 20 mM MES and 0.5 mM Na_2_EDTA, pH 7.0. Abbreviations: **C**^**L**^ – LNA cytidine, **s**^**2**^**U** – 2-thiouridine, ^*^ΔK_d_ – standard deviation based on three independent measurements.

The most favorable K_d_ value was obtained for the T4 triplex, which contains five LNA and six 2-thioU residues (K_d_ = 2.47 ± 0.51 µM, Table 2). In contrast, the T3 triplex with the same number of LNA and 2-thioU residues, but they were placed at different positions and resulted in the lowest K_d_ value (K_d_ = 45.2 ± 9.04 µM, Table 2). Moreover, the binding affinity of the TFO with three LNA and four 2-thioU moieties was similar to the affinity of the TFO variant with three LNA and two 2-thioU residues (K_d_ = 3.01 ± 0.24 µM vs. 3.94 ± 0.47 µM, T1 vs. T7, respectively, Table 2). Unfortunately, we did not find a simple correlation between the number of LNA and 2-thioU substitutions within the TFO strand and the values of the K_d_ parameter for all model triplexes. However, a correlation between the position of the modified nucleotides and the thermodynamic stability within the RNA duplexes was reported^10^. Currently, it is difficult to explain why the triplexes that contain more modified residues are not characterized by a more favorable dissociation constant in comparison to the variants with fewer LNA and 2-thioU residues. Presumably, not only the presence and number of these modifications have an influence on TFO-hairpin interaction specificity, but their position within the TFO strand is also important, which could be the effect of TFO structure and ability of docking to double-stranded region^3^. The presence of modifications such as LNA and 2-thioU within TFO is absolutely necessary to make RNA triplexes stable at pH 7.0. These modifications preorganize single stranded structure of TFO to make binding to hairpin stem thermodynamically more favorable^12^. On the other side too much rigid TFO structure makes that binding less favorable. Pabon-Martinez *et al*. confirmed that reduced number of LNAs on consecutive positions at the 3′-end of TFO decreases the rate of triplex formation. In contrast, the LNAs at 5′-end in TFO result in more efficient triplex formation^16^.

Moreover, the dissociation constant values of different RNA triplexes might be useful for the detailed study of triplexes, for instance their interactions within a cell. The EMSA data showed that the dissociation constant values of model triplexes fall in the range of low micromolar concentrations, under the conditions comparable to physiological ones. These K_d_ values are in the range reported by other investigators for RNA and DNA triplexes^40,41^. Considering the specificity of LNA and 2-thioU modified TFOs, as well as hairpin interactions, we decided to test their potential in *HeLa* cells. The *HeLa* cells environment encompasses potassium and magnesium cations as well. To evaluate the influence of both cations on the triplex stability, we performed EMSA analysis for T2 triplex in MES buffer containing 90 mM potassium chloride, 10 mM sodium chloride and 0.4 mM magnesium chloride (pH 7.0)^43^. Under these conditions, K_d_ value was 1.2 μM, whereas under the standard conditions (100 mM sodium chloride), K_d_ value was 27.3 μM for T2 triplex (Supplementary Fig. S2). This observation suggests that the presence of both cations stabilizes the RNA triplex formation. A similar effect for DNA triplexes has been previously reported^44,45^.

### The effect of RNA triplex structures on GFP gene expression

Recently, extensive biological studies have shown that both DNA and RNA TFOs represent an attractive tool for gene expression regulation^32,46,47^. TFOs have been shown to inhibit transcription *in vitro* and the expression of target genes in cell culture by the formation of a triplex structure with genomic DNA^31^. Li *et al*. investigated the triplex formation by targeting long noncoding RNAs to DNA, and proposed a general mechanism of this process^20^.

Due to the pH-dependence of triplex formation, it is important to increase its stability in the natural environment of the cell (at neutral pH), thus improving its potential for *in vivo* applications. Hence, chemically modified RNA triplexes (T1–T8) were searched for their modulation of reporter gene expression in the *HeLa* cell line because previous thermodynamic studies revealed that these structures are stable under near physiological conditions. In addition, the specificity of the studied RNA triplex interactions (EMSA experiments) allows for applying the TFOs containing LNA and 2-thioU modifications in a cellular context. The effect induced by TFOs with LNA and 2-thioU residues (eight variants) was determined using the quantitative polymerase chain reaction (qPCR) technique.

The GFP gene expression studies in *HeLa* cells were conducted with pZsGreen-N1 plasmid construct, carrying RNA triplex-forming target sequence. We first examined the possibility of interactions between the TFO variants and pZsGreen-N1 plasmid with a target insert. As expected, our results show a very low (8–10%) binding affinity of the TFO and pZsGreen-N1 vector, even at hundred fold molar excess of TFO (Supplementary Fig S3A). Furthermore, we performed the gel competitive binding assay to test the binding of TFO2 and TFO5 to the RNA hairpin in the presence of pZsGreen-N1 plasmid construct. The resulting gel shows the bands which correspond to the formation of T2 and T5 triplexes, even when plasmid was added (Supplementary Fig S3B). The data indicate the specificity of TFOs and RNA hairpin interactions. Together, these observations and RNA overexpression character of pZsGreen-N1 plasmid make a double-stranded DNA as a very unlikely target for TFO. Moreover, these results suggest that a decrease in GFP expression should occur due to inhibition at the RNA level.

**Figure 4:**
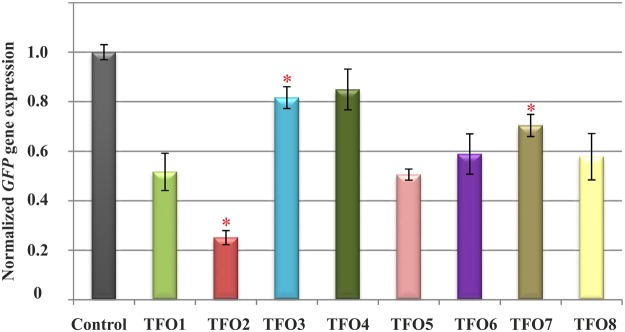
The effect of triplex forming oligonucleotides (TFO1-TFO8) on GFP gene expression in *HeLa* cell line. Statistically significant differences in the mean expression between control and modified TFOs (P < 0.05) were marked with an asterisk on the charts presenting the qPCR results.

*HeLa* cells carrying pZsGreen-N1 plasmid construct were transfected with 150 nM concentration of TFO1-TFO8. As a control was used a TFO which is unable to form triplex with the target hairpin and did not affect assay. After transfection the qPCR was used to analyze TFOs effect. Figure 4 demonstrates the results of the analysis of TFO-mediated inhibition of GFP gene expression.

The data obtained from these studies show that all variants of TFO cause downregulation of gene expression (Fig. 4). Moreover, the results demonstrate differences of GFP gene silencing between particular TFO variants. Interestingly, the expression of GFP was significantly reduced in the presence of TFO2 in comparison to the control TFO (75%, Fig. 4). Moreover, the level of GFP gene expression was found to be reduced by 18%, 15% and 30% in TFO3, TFO4 and TFO7 treated cells, respectively, whereas the inhibition of gene expression was more efficient for TFO1, TFO5, TFO6 and TFO8 compared to the control TFO (48%, 49%, 41% and 42%, respectively, Fig. 4). These observations reflect the incorporation of modified nucleotides into the TFO strand. In addition, the number and position of LNA and 2-thioU nucleotides affect the biological potential of TFOs^48^. Despite the lack of direct correlation between the binding affinities of TFOs and their GFP gene silencing, simultaneous incorporation of the two modifications (LNA and 2-thioU) into TFO strand is beneficial for triplex formation. The ability of modified TFOs to selectively modulate the activity of genes is a promising alternative in diagnostics and therapy.

In conclusion, the results presented herein provide information about the thermodynamics, structural and biological properties of model triplexes. The obtained data demonstrate that LNA and 2-thioU modifications are beneficial for triplex formation and increase the resistance of TFOs within the cellular environment. In summary, our findings expand our knowledge of RNA triplex structures containing LNA and 2-thioU residues. The behavior of modified nucleotides makes them an alternative chemical tool for designing triplexes with improved stability under physiological conditions.

## Methods

### Oligonucleotides synthesis

All oligonucleotides were synthesized on a MerMade12 (BioAutomation) synthesizer using standard phosphoramidite chemistry^49^. FAM-labeled oligonucleotides were synthesized using fluorescein-labeled phosphoramidite. Thin-layer chromatography (TLC) purification of oligonucleotides of length 12 nt was performed on Merck 60 F254 TLC plates with 1-propanol/ammonia/water = 55:35:10 (v/v/v). Oligonucleotides of length 32 nt were purified by polyacrylamide gel electrophoresis. The details of deprotection and purification of oligonucleotides have been described previously^50^. Purified oligonucleotides were characterized using MALDI-TOF mass spectrometry.

### UV melting experiments

Oligonucleotides were dissolved in a buffer containing 100 mM sodium chloride, 20 mM MES and 0.5 mM Na_2_EDTA, pH 7.0. Oligonucleotide single strand concentrations were calculated based on the absorbance measured above 80 °C and extinction coefficients, which were approximated by a nearest-neighbor model using the ribotask.com website. LNA and 2-thioU modified and unmodified RNA strands with identical sequences were assumed to have identical extinction coefficients. The samples were renatured for 3 min at 90 °C and then cooled to room temperature overnight. The measurements were performed for nine different concentrations of each triplex in the concentration range 10^−4^–10^−6^ M. Absorbance versus temperature curves were obtained by the UV melting method at 260 nm in the temperature range 4–90 °C with a heating rate of 0.5 °C/min on a Jasco V-650 spectrophotometer equipped with a thermoprogrammer. Melting curves were analyzed, and the thermodynamic parameters were determined by non-linear curve fitting with MeltWin 3.5 software. Melting temperatures calculated for 10^–4^ M oligonucleotide concentration are marked by T_m_.

### Circular dichroism measurements

CD spectra were recorded on a Jasco J-815 spectropolarimeter using 1.5 ml quartz cuvettes with a 5 mm path length. The oligonucleotides were dissolved in a buffer containing 100 mM sodium chloride, 20 mM MES and 0.5 mM Na_2_EDTA, pH 7.0 to achieve a 20 µM sample concentration. All samples were renatured for 3 min at 90 °C and then slowly cooled to room temperature overnight before data collection. The measurements were taken at 10 °C in the 210–320 nm wavelength range with a 1 nm data interval. The CD curves were established as an average of three CD measurements. The buffer spectrum was subtracted from the sample spectra.

### Electrophoretic mobility shift assay (EMSA)

The 1 µM of FAM labeled RNA hairpin was mixed with increasing concentration of LNA and 2-thioU modified TFO. Oligonucleotides were dissolved in a buffer containing 100 mM sodium chloride, 20 mM MES and 0.5 mM Na_2_EDTA, pH 7.0 to obtain final volume of 10 µl. The samples were renatured by 3 min at 90 °C and then cooled to room temperature overnight. After folding, 2 µl of 35% glycerol solution was added. Samples were centrifuged and loaded into a 12% native polyacrylamide gel prepared in 1X TAE buffer, pH 7.0. The electrophoresis was performed at 10 W for 6.5 h at room temperature. The results obtained from three independent EMSA experiments. The data analysis was performed with Origin 8.0 software and the triplex dissociation constant (K_d_) was determined from the concentration of TFO that caused half of the hairpin to form a triplex and was calculated using the least squares algorithm^39^.

### *HeLa* cell line assays and qPCR statistical analysis

*HeLa* cell experiments were performed with the use of pZsGreen-N1 expression plasmid with the GFP protein. The PCR products (carrying RNA hairpin sequence) and the pZsGreen-N1 vector were digested by EcoRI and PstI restriction enzymes, at 37 °C overnight. The products of digestion were verified by electrophoresis on agarose gel, and then purified. Next, the products were ligated by T4 DNA ligase (Thermo Scientific), at a 3:1 ratio, at 4 °C overnight. The 130-nucleotide fragment of the sequence of interest (Supplementary Materials) was inserted into a pZsGreen-N1 expression plasmid, upstream of the GFP protein coding region. The recombinant plasmid was cultured in *E*. *coli* in LB medium with kanamycin, overnight at 37 °C. The pZsGreen-N1 plasmid construct was extracted with the Qiagen extraction kit (Qiagen Plasmid Midi Kit 25), and the construct was then confirmed through sequencing.

One day before transfection, *HeLa* cells were passaged to 24-well plates and cultured in a standard RPMI 1640 medium containing 10% FBS (Invitrogen), 1x Antibiotic-Antimycotic solution (Sigma Aldrich) and 1x MEM vitamins solution (Sigma Aldrich). When the confluence reached ca. 90% per well, co-transfection of the pZsGreen-N1 plasmid constructs of 1 μg and TFOs at concentrations of 150 nM was carried out with Lipofectamine 2000 (Invitrogen) in medium without antibiotics. After 24 h incubation, cells were washed with phosphate-buffered saline (PBS) and then total RNA was extracted. Each transfection experiment (using various TFOs) was repeated at least three times. For quantitative analysis of silencing, total RNA from cells was isolated using the TRIzol method^51^. Next, 0.5 μg of RNA was subjected to DNase I (Life Technologies) treatment. RNA quality was controlled by separation on 1.5% agarose gels. cDNA, which was the template for qPCR, was obtained from a reverse transcription reaction using iScript cDNA Synthesis Kit (Bio-rad). qPCR was performed on a CFX96 real-time PCR system (Bio-Rad) using iTaq SYBR Green Supermix (Bio-rad) and 96-well clear plates. The level of GFP mRNA was quantified with the use of target gene primers which are listed in Supplementary Materials. Statistical analysis of the qPCR results was performed with Bio-Rad CFX Manager 3.0 and OriginLab 8.0 software. The results from the biological replicates for particular samples were gathered to determine the mean relative expression and its standard deviation (Bio-Rad CFX Manager 3.0). Next, the normalized expression of the GFP gene was compared using a significance level of 0.05 for the particular analyzed TFOs.

## Electronic supplementary material

Supplementary Materials
