# Supplementary material for: Modified RNA triplexes: Thermodynamics, structure and biological potential
Source: Sci Rep. 2018 Aug 29;8:13023. doi: 10.1038/s41598-018-31387-5 (PMC6115336; doi:10.1038/s41598-018-31387-5)
Supplement: Supplementary file 1 — Supplementary Materials [file 41598_2018_31387_MOESM1_ESM.doc]

**Supplementary Materials**

**Modified RNA triplexes: Thermodynamics, structure and biological potential**

Marta Szabat, Elzbieta Kierzek, Ryszard Kierzek^1^

Institute of Bioorganic Chemistry, Polish Academy of Sciences, Noskowskiego 12/14, 61-704 Poznan, Poland

^1^Correspondence to: [rkierzek@ibch.poznan.pl](mailto:rkierzek@ibch.poznan.pl); *Tel:+* 48 618 528 503*; Fax:+48* 618 520 532

**The 130-nucleotide fragment of the target sequence**

5'-TATTATATATGAATTCTAATACGACTCACTATAGGGTACGTGCTAGTCACATGCATGCTAGAGAGAGAAAGTTTCGACTTTCTCTCTCTATACGTGCATTACTGACATGCATGCGACGTCTATATATATT

**The sequence of the primers for qPCR analysis**

GFPf 5'-GTACCACGAGTCCAAGTTCTAC

GFPr 5'-CCAGTTGTCGGTCATCTTCTT

**Supplementary Figures**


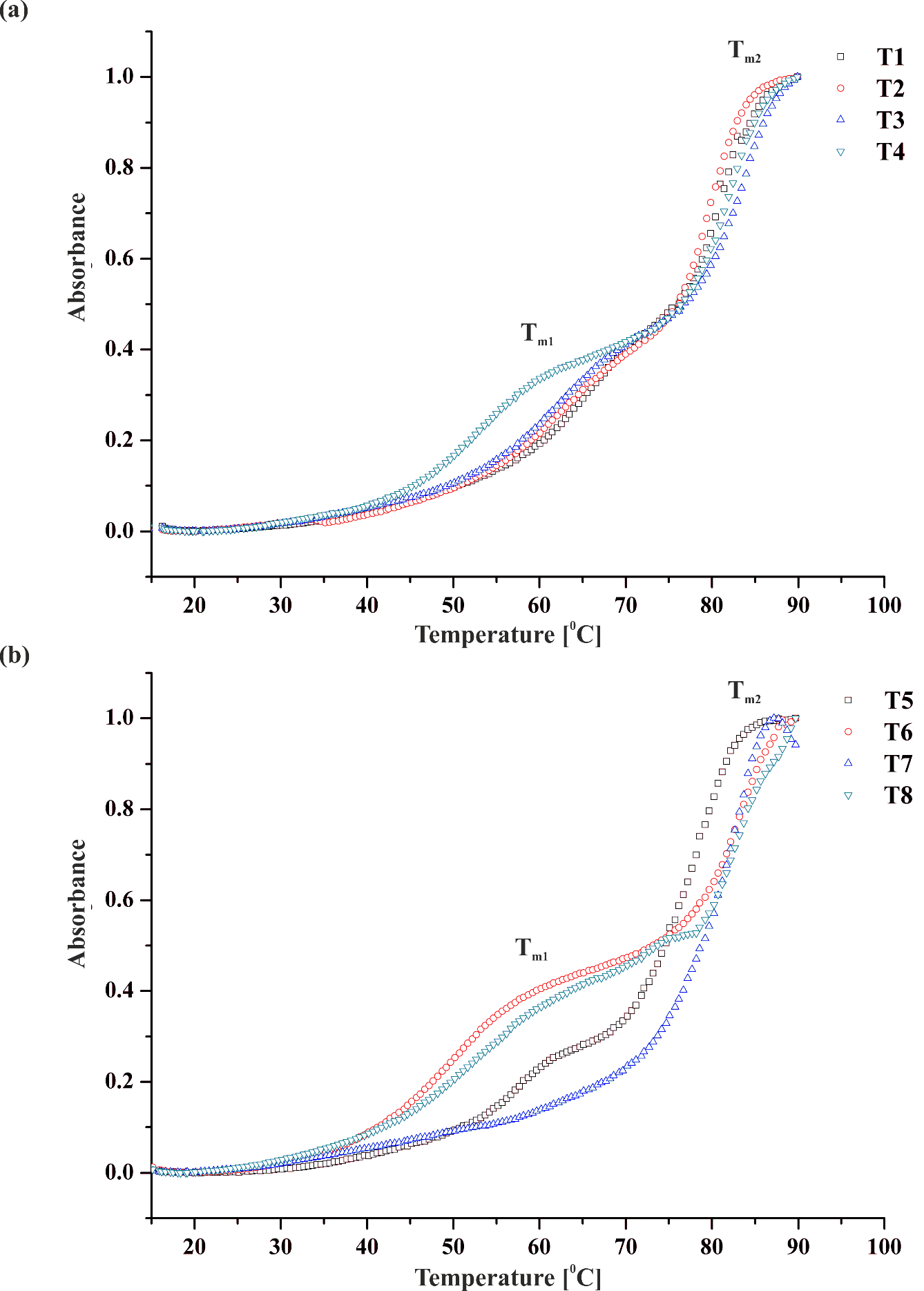


**Figure S1.** The melting curves of model T1-T4 (a) and T5-T8 (b) triplexes. Tm_1_ and Tm_2_ - melting temperatures of triplex and RNA hairpin, respectively.

**
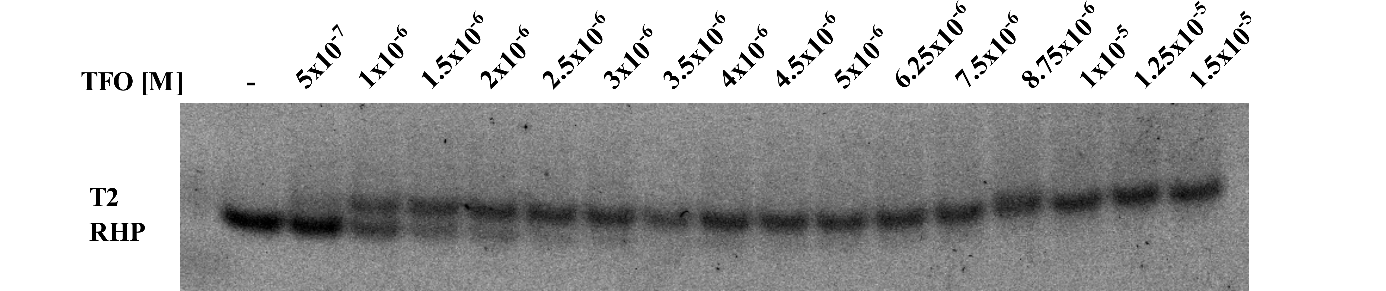
**

**Figure S2.** Electrophoretic mobility shift assays of T2 triplex in MES buffer containing 90 mM potassium chloride, 10 mM sodium chloride and 0.4 mM magnesium chloride (pH 7.0). Electrophoresis was performed in 1X TAE buffer, pH 7.0 supplemented with 90 mM KCl, 10 mM NaCl, 0.4 mM MgCl_2_. The 1 µM of FAM labeled RNA hairpin was mixed with increasing concentration of TFOs.

**
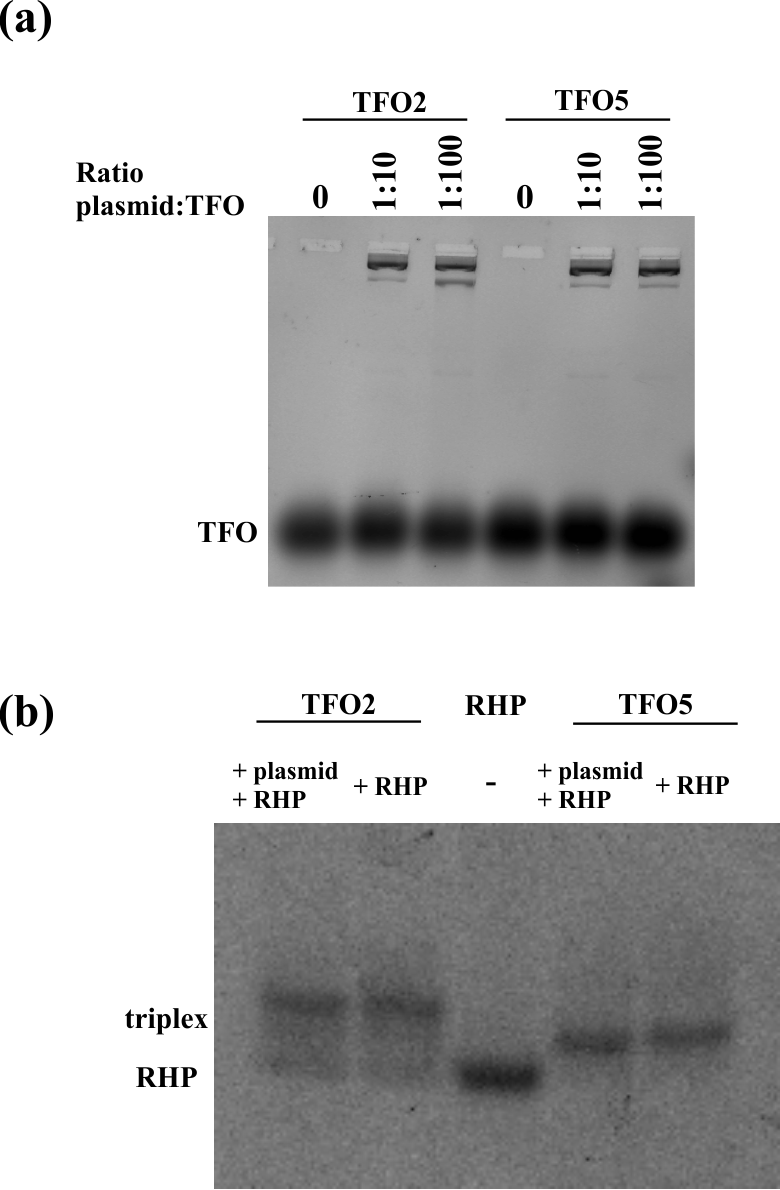
**

**Figure S3.** Influence of pZsGreen-N1 plasmid with target insert on triplex formation between TFO and RNA hairpin. (a) Agarose gel electrophoresis showing the TFO2 and TFO5 binding of pZsGreen-N1 plasmid with target insert. The concentrations of FAM labeled TFO were 10 and 100 µM, corresponding to the following ratio of plasmid versus TFO, 1:10 and 1:100, respectively. (b) Polyacrylamide gel electrophoresis showing the TFO2 and TFO5 binding of RNA hairpin in the presence of pZsGreen-N1 plasmid with target insert. The 1 µM of FAM labeled RNA hairpin was used. The concentrations of TFO and plasmid were 10 and 1 µM, respectively. Both electrophoresis were performed in MES buffer containing 90 mM potassium chloride, 10 mM sodium chloride and 0.4 mM magnesium chloride (pH 7.0).
